# Dynamical behaviour of ultrathin [CoFeB (*t*_*CoFeB*_)/Pd] films with perpendicular magnetic anisotropy

**DOI:** 10.1038/s41598-020-79632-0

**Published:** 2021-01-08

**Authors:** Ana S. Silva, Simão P. Sá, Sergey A. Bunyaev, Carlos Garcia, Iñigo J. Sola, Gleb N. Kakazei, Helder Crespo, David Navas

**Affiliations:** 1 Departamento de Fisica e Astronomia, Faculdade de Ciências, Institute of Physics for Advanced Materials, Nanotechnology and Photonics (IFIMUP), Universidade do Porto, 4169-007 Porto, Portugal; 2 Departamento de Física y Centro Científico Tecnológico de Valparaíso-CCTVal, Universidad Técnica Federico Santa María, 2390123 Valparaíso, Chile; 3 Laser Applications and Photonics Group, Applied Physics Department, University of Salamanca, 37008 Salamanca, Spain; 4 Instituto de Ciencia de Materiales de Madrid, ICMM-CSIC, 28049 Madrid, Spain

**Keywords:** Materials science, Nanoscience and technology, Physics

## Abstract

CoFeB-based ultrathin films with perpendicular magnetic anisotropy are promising for different emerging technological applications such as nonvolatile memories with low power consumption and high-speed performance. In this work, the dynamical properties of [CoFeB (*t*_*CoFeB*_)/Pd (10 Å)]_5_ multilayered ultrathin films (1 Å ≤ *t*_*CoFeB*_ ≤ 5 Å) are studied by using two complementary methods: time-resolved magneto-optical Kerr effect and broadband ferromagnetic resonance. The perpendicular magnetization is confirmed for multilayers with *t*_*CoFeB*_ ≤ 4 Å. The effective perpendicular magnetic anisotropy reaches a clear maximum at *t*_*CoFeB*_ = 3 Å. Further increase of CoFeB layer thickness reduces the perpendicular magnetic anisotropy and the magnetization became in-plane oriented for *t*_*CoFeB*_ ≥ 5 Å. This behaviour is explained by considering competing contributions from surface and magnetoelastic anisotropies. It was also found that the effective damping parameter *α*_*eff*_ decreases with CoFeB layer thickness and for *t*_*CoFeB*_ = 4 Å reaches a value of ~ 0.019 that is suitable for microwave applications.

## Introduction

Since the mid 1970s ^1,2^, materials with perpendicular magnetic anisotropy (PMA) have been studied for a large diversity of applications including, but not limited to, perpendicular recording media^1–4^, patterned magnetic media^5,6^, high-density spin-transfer torque magnetic random access memories (STT-MRAM)^7–9^ and more recently in skyrmion-based devices^10–12^ and synthetic antiferromagnets for biomedical applications^13–15^.

Among different materials, CoFeB-based thin films have received great attention since 2010, when Ikeda et al*.*^9^ demonstrated that this alloy in contact with a MgO layer can show PMA. This behaviour can be achieved when the CoFeB layer is thin enough, and the CoFeB/MgO interfacial anisotropy contribution^9,16,17^ overcomes the volumetric terms of the anisotropy energy^18^. Afterwards, several works have been focused on the optimization of the PMA in CoFeB thin films by using different materials for the capping and/or buffer layers^19^, including Ta^20,21^, Hf^22,23^, Mo^24,25^, Ru^26,27^, V^26^, Nb^27^, Pt^28,29^, Pd^30–32^ and Au^33^.

Moreover, CoFeB alloys show higher spin polarization than Co, Fe and Ni (up to 65% vs. around 45%)^34^, and can be grown with a tuned damping parameter (*α*)^9,35^. Since damping determines the temporal performance of magnetic devices such as the timescale for magnetization reversal or spin-transfer-torque (STT) switching, *α* is a key parameter for the development of several technological applications. In particular, materials with low *α* have been suggested for high-speed spintronic devices, such as in STT-based systems with low switching and power consumption^8,36^, as well as in magnetic tunnel junctions (MTJs) with high signal-to-noise ratio^9^, and in magnonic devices^37^. On the other hand, systems with high α values have been suggested for spin pumping and inverse spin Hall effect applications^38–40^. Therefore, understanding the behaviour of the α parameter is of particular importance.

Although it was reported that the Gilbert damping (*α*_*Gilbert*_) for Co_40_Fe_40_B_20_ alloy can be as low as 0.004^41,42^, the estimated effective damping (*α*_*eff*_) increases substantially at low thickness^9^ which is detrimental to the development of STT- and MTJs-based devices. For example, it was reported that 1 or 1.3 nm thick CoFeB layers with PMA show *α*_*eff*_ parameters of 0.012^35^ and 0.027^9^, respectively. This rise has been associated with the fact that the *α*_*eff*_ parameter contains contributions from both intrinsic and extrinsic terms^43^. The intrinsic term or *α*_*Gilbert*_ is constant with the resonance frequency but shows temperature dependence^43,44^. Devolder et al*.*^35^ studied the correlation between *α*_*Gilbert*_ and the g-factor (*g*) in CoFeB thin films as a function of the alloy composition and annealing conditions. As it was expected in transition metals, the dependence *α*_*Gilbert*_ *≈* (*g* − 2)^2^ was reported^45^.

On the other hand, it has been suggested that the extrinsic term can be due to different effects such as^46^:

1. An inhomogeneity contribution based on the local variations of the magnetization and/or the magnetic anisotropy field due to structural defects and/or thickness variations^43^. In particular, Devolder et al.^35^ studied the damping parameter of Co_x_Fe_80−x_B_20_ ultrathin films and reported that the inhomogeneity term should be constant with the resonance frequency while it should depend on the sample composition, thickness, and materials used for the capping and/or buffer layers.
2. A two-magnon scattering (TMS) contribution^47,48^. Regarding this term, Liu et al*.*^41^ performed out-of-plane angular dependence FMR measurements of CoFeB thin films, and they reported that the damping mechanism depends on the layer thickness. It was suggested that *α*_*eff*_ is mainly governed by the Gilbert damping in thicker (≥ 4 nm) CoFeB films, while inhomogeneous broadening and two-magnon scattering are the main factors for films thinner than 2 nm.
3. A contribution from the spin-pumping effect^49,50^. Although this effect was first suggested by Berger^51^, it was experimentally confirmed by Mizukami et al*.*^52,53^ that the damping of non-magnetic/magnetic/non-magnetic trilayers depends on the non-magnetic material. Large damping parameters were determined when non-magnetic materials with a strong spin–orbit coupling, such as Pt and Pd, were used. Iihama et al*.*^20^ investigated the damping parameter of Ta/CoFeB/MgO and Ta/CoFeB/Ta thin films using an all-optical pump-probe method, and they claimed that the enhancement of *α*_*eff*_ is caused by the spin pumping effect at the Ta/CoFeB interfaces.
4. Any change in the magnetization of a ferromagnetic material placed on top of a coplanar waveguide (CPW) induces eddy currents. These eddy currents generate a magnetic field in the ferromagnet that opposes the original change and provides a damping mechanism (*α*_*eddy*_)^54,55^. Moreover, the generated eddy currents also affect the CPW, and an extra damping mechanism, known as radiative damping (*α*_*rad*_), should be considered^56,57^.

Although CoFeB/Pd multilayers with strong PMA were first reported in 2010^30,31^, subsequent works have been mainly focused on understanding the contributions of the volumetric (*K*_*v*_) and surface (*K*_*s*_) anisotropy terms to the PMA as a function of the CoFeB layer thickness and the number of CoFeB/Pd bilayers^32,58,59^. In this paper, we have studied the dynamical behaviour of ultrathin [CoFeB (*t*_*CoFeB*_)/Pd (10 Å)]_5_ films with CoFeB thicknesses ranging from 1 to 5 Å by using both vector network analyzer based ferromagnetic resonance (VNA-FMR) and time-resolved magneto-optical Kerr effect (TR-MOKE) measurements. We observed that PMA was achieved for *t*_*CoFeB*_ ≤ 4 Å, and both *K*_*v*_ and *K*_*s*_ were estimated. In addition, we have performed a systematic study of previously unreported characteristic magnetic parameters such as the CoFeB saturation magnetization and the damping parameter in ultrathin CoFeB/Pd films. Moreover, the effective damping parameter and its related intrinsic and extrinsic contributions were analyzed as a function of *t*_*CoFeB*_.

## Results and discussion

**Figure 1:**
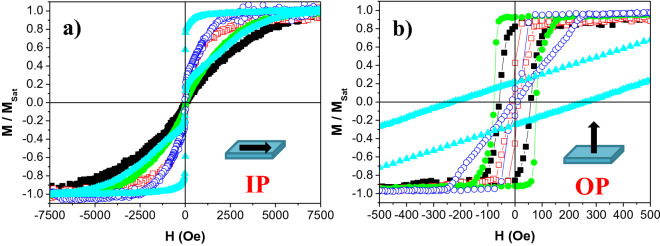
In-plane (**a**) and out-of-plane (**b**) hysteresis loops of [CoFeB (*t*_*CoFeB*_)/Pd (10 Å)]_5_ multilayer thin films with *t*_*CoFeB*_ = 1 (Black filled square), 2 (red open square), 3 (green filled circle), 4 (dark blue open circle) and 5 Å (light blue filled triangle).

Figure 1a and b show the in-plane and out-of-plane hysteresis loops of the [CoFeB (*t*_*CoFeB*_)/Pd (10 Å)]_5_ multilayer stacks, respectively. The loops indicated that our samples show PMA when the CoFeB thickness (*t*_*CoFeB*_) ranged from 1 to 4 Å. However, the easy magnetization axis lays in-plane for the largest *t*_*CoFeB*_ (5 Å).

### Magnetic moment

**Figure 2:**
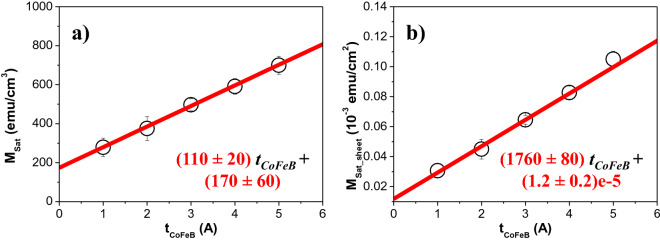
(**a**) Saturation magnetization, *M*_*Sat*_ as a function of the CoFeB layer thickness (*t*_*CoFeB*_*)*. (**b**) *M*_*Sat_sheet*_ as a function of the CoFeB layer thickness (*t*_*CoFeB*_*)*. The continuous red lines are linear fits.

Moreover, the hysteresis loops were used to obtain the values of the saturation magnetization (*M*_*Sat*_), which are shown in Fig. 2a. We observed that *M*_*Sat*_ increases with the CoFeB film thickness from (280 ± 50) to (700 ± 50) emu/cm^3^ for *t*_*CoFeB*_ = 1 and 5 Å, respectively. This behavior was already observed in ultrathin films, such as for example in Pt*/*Co/Pt^60^, Ta/CoFeB (*t* nm)/SiO_2_ (*t* = 1, 2, 3, 4, 6, 10 13 nm)^61^, [Co (*t* nm)/Pd]_8_^62^ and [CoFeB (*t* nm)/Pd (1.0 nm)]_10_ (*t* = 0.4, 0.6, 0.8, 1.0 and 1.2 nm) multilayer films^32^ with perpendicular anisotropy.

As the CoFeB thickness was varied, while both the Pd thickness (1 nm) and the number of CoFeB/Pd bilayers (5) were kept constant, the rise of *M*_*Sat*_ with the CoFeB film thickness could be associated with the increase of the volume magnetic moment contribution while the surface/interface magnetization term should remain constant^32^. Therefore, the experimental magnetization data can be fitted by a linear dependence (the continuous line in Fig. 2a) and the surface/interfacial magnetization term can be estimated from the extrapolation of this linear dependence to zero thickness. Our analysis determined that the surface/interfacial magnetization of CoFeB/Pd interfaces is (170 ± 60) emu/cm^3^, which agrees with the value of (180 ± 10) emu/cm^3^ reported by Ngo et al.^32^.

In addition, the magnetization per unit area (*M*_*Sat_sheet*_) vs. the CoFeB film thickness (*t*_*CoFeB*_) is shown in Fig. 2b. As described by Engel et al*.*^63^, *M*_*Sat_sheet*_ in superlattices can be estimated from

1 $$M_{Sat\_sheet} = I/\left( {N \times A} \right) = M_{eff\_CoFeB}\times t_{CoFeB} + M_{eff\_Pd}\times t_{Pd}$$

where *I* is the measured magnetic moment, *N* is the number of bilayers, *A* is the area of the films (determined from the software analysis of digital photographs of the samples), *M*_*eff_CoFeB*_ is the effective CoFeB saturation magnetization, *t*_*CoFeB*_ is the CoFeB layer thickness, *M*_*eff_Pd*_ is the effective magnetization of the polarized Pd induced by the ferromagnetic proximity effect^63–65^, and *t*_*Pd*_ is the Pd layer thickness (10 Å). A linear fit of (*M*_*Sat_sheet*_ vs. *t*_*CoFeB*_) is also plotted in Fig. 2b. Assuming that the magnetic moment is uniformly induced in the entire Pd layer, *M*_*eff_Pd*_ = (120 ± 20) emu/cm^3^ was estimated from the extrapolation of the linear fit to zero CoFeB layer thickness (*t*_*CoFeB*_ = 0 Å). This saturation magnetization is in good agreement with previous studies on Co/Pd multilayers^64–66^. Additionally, an effective CoFeB saturation magnetization value of *M*_*eff_CoFeB*_ = (1760 ± 80) emu/cm^3^ was obtained from the linear fit slope.

This value seems to be too large in comparison with the values previously reported for Co_40_Fe_40_B_20_ thin films and estimated from the hysteresis loops (1000^30^ or 1034 emu/cm^3^^31,67^), ferromagnetic resonance measurements (1019 emu/cm^3^^68^) or the linear fit of the magnetization per unit area (*M*_*Sat_sheet*_) vs the CoFeB film thickness (1120^69^, 1150^25^ or 1200 emu/cm^3^^26^). Usually, such large *M*_*sat*_ values have been reported for CoFeB alloys fabricated with lower B concentration, such as ≈ 1670 and 1830 emu/cm^3^ for (Co_35_Fe_65_)_90_B_10_ and (Co_35_Fe_65_)_87.5_B_2.5_ respectively^70^, or in CoFeB multilayers in which the boron atoms diffused out of the CoFeB alloy due to the application of an annealing process (1900 emu/cm^3^ for Ta/Co_40_Fe_40_B_20_/MgO multilayers)^71^. However, in 2013, Sinha et al*.*^72^ studied the perpendicular magnetic anisotropy in Ta/CoFeB (*t* nm)/MgO multilayers with CoFeB thickness ranging from 0.8 to 5 nm. In agreement with the literature, they reported that the linear fit slope of (*M*_*Sat_sheet*_ vs. *t*) provides an effective CoFeB saturation magnetization of *M*_*eff_CoFeB*_ = 1210 emu/cm^3^ for thicker samples (*t* ≥ 2.2 nm). But large *M*_*eff_CoFeB*_ values, such as 1790 emu/cm^3^, were determined for the thinnest CoFeB layers (*t* ≤ 2.2 nm). Therefore, it was suggested that the effective saturation magnetization increases from its bulk value below a certain magnetic layer thickness. This behaviour was confirmed in our work as well as in Ref.^32^, where Ngo et al*.* reported *M*_*eff_CoFeB*_ = 1550 emu/cm^3^ in CoFeB (*t* nm)/Pd multilayered thin films.

### Anisotropy energy terms

The origin of PMA in ultrathin multilayer stacks, such as (CoFeB/Noble metal) bilayers, is based on the competition between the volumetric (*K*_*v*_) and surface (*K*_*s*_) terms of the anisotropy energy^18^. To distinguish between both contributions, we performed ferromagnetic resonance measurements when the DC external field was applied perpendicular to the sample plane. In general, the FMR spectrum of a thin film depends significantly on the presence of different anisotropies, such as the magnetocrystalline, shape, magnetoelastic and surface contributions, and it can be described through the Kittel equation^20,73,74^:

2 $$f_{FMR} = \frac{\gamma }{2\pi }\sqrt {\left( {H\cos \left( {\theta_{0} - \theta_{H} } \right) + H_{eff} \cos^{2} \left( {\theta_{0} } \right)} \right) \times \left( {H\cos \left( {\theta_{0} - \theta_{H} } \right) + H_{eff} \cos \left( {2\theta_{0} } \right)} \right)}$$

where (*γ/2π*) = (*gµ*_0_/*h*) is the gyromagnetic ratio; *g* is the g-factor; *µ*_0_ is the Bohr magneton; *h* is Planck's constant; *H* is the external applied DC magnetic field; *H*_*eff*_ is the effective anisotropy field, that is positive for the films with perpendicular magnetization in the absence of external magnetic field and negative for films with in-plane magnetization; *θ*_*H*_ is the angle of the external applied magnetic field and *θ*_0_ is the equilibrium angle of the sample magnetization. The equilibrium angle *θ*_0_ can be derived from the equation:

3 $$H\sin \left( {\theta_{H} - \theta_{0} } \right) = \frac{1}{2}H_{eff} \sin \left( {2\theta_{0} } \right)$$

When thin films are saturated and *θ*_*H*_ = *θ*_*0*_, Eq. (2) can be reduced to^74^:

4 $$f_{FMR} = \frac{\gamma }{2\pi }\left( {H + H_{eff} } \right)\quad {\text{for perpendicular geometry}}$$

**Figure 3:**
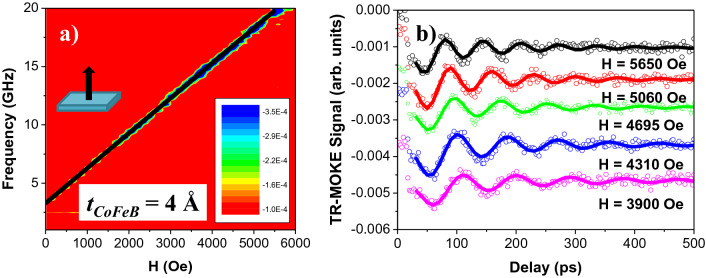
(**a**) VNA-FMR spectra of the [CoFeB (4 Å)/Pd (10 Å)]_5_ multilayer thin film with the external magnetic field applied perpendicularly to the sample plane. The black line corresponds to the fits using the Kittel formula (Eq. 4). (**b**) Time-resolved magneto-optical Kerr effect (TR-MOKE) signals of a [CoFeB (2 Å)/Pd (10 Å)]_5_ multilayer thin film under different external applied fields (H = 5650, 5060, 4695, 4310 Oe and 3900 Oe) and when *H* was applied at *θ*_*H*_ = 78°. Theoretical curves (solid curves) are fits to the experimental data (open symbols) using Eq. (5). Data was moved along the vertical axis for clarity.

Figure 3a shows the perpendicular VNA-FMR spectrum of the [CoFeB (4 Å)/Pd (10 Å)]_5_ multilayer thin film with PMA as well as a black line, which corresponds to the fits using the Kittel formula (Eq. 4).

However, we should note that we were not able to achieve a good signal-to-noise ratio from samples with the thinnest CoFeB thicknesses (*t*_*CoFeB*_ = 1–3 Å). For these samples, our analyses were complemented by performing TR-MOKE measurements. All-optical pump–probe technique has been successfully used for understanding the dinamical response of the ultrafast magnetization, the magnetization precession and the effective damping, in materials with perpendicular anisotropy such as [Co/Pt]_n_^75,76^ and [Co/Pd]_8_^62^ multilayers, L1_0_-FePt alloy epitaxial thin films^73^ or Ta/CoFeB/MgO(Ta) thin films^20^. As an example, Fig. 3b shows the TR-MOKE signals for [CoFeB (2 Å)/Pd (10 Å)]_5_ and as a function of the external applied magnetic field. An ultrafast demagnetization process on the subpicosecond timescale is observed after the application of the pump pulse, followed by a quick remagnetization stage that shows a precessional response in the last section. Both the ferromagnetic resonance frequency (*f*_*FMR*_) and the damping parameter (*α*) characterize the oscillatory response of the magnetization and it can be fitted by^73,77^:

5 $$\theta = \theta_{0} + A \times e^{{ - t/t_{0} }} + B \times \sin \left( {2\pi f_{FMR} t + \varphi } \right) \times e^{ - t/\tau }$$

where *θ*_*0*_ and *A* are the background magnitudes, and *t*_*0*_ is the background recovery time. The final term represents the precessional motion where *B*, *f*_*FMR*_, *φ* and *τ* are the amplitude, frequency, phase, and relaxation time, respectively. A good agreement between the model and the experimental data is shown in Fig. 3 (b) and the fits of the *f*_*FMR*_ field-dependence were performed through the set of Eqs. (2) and (3)^20,73^.

The main results of our VNA-FMR and TR-MOKE fits are summarized in Table 1. The gyromagnetic ratio and g-factor did not show any significant dependence with the CoFeB thickness, and we obtained average values of (2.99 ± 0.03) GHz/kOe and (2.14 ± 0.03), respectively, in agreement with the literature^23,35,68,78^.

**Figure 4:**
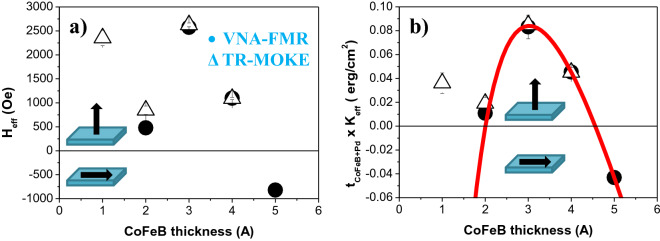
(**a**) *H*_*eff*_ and (**b**) (*t*_*CoFeB*+*Pd*_ × *K*_*eff_CoFeB*_) as a function of the CoFeB layer thickness (*t*_*CoFeB*_ = 1 – 5 Å) for [CoFeB (*t*_*CoFeB*_)/Pd (10 Å)]_5_ multilayer thin films. While black open circle are the experimental data determined from the VNA-FMR measurements, black open triangle were determined from the TR-MOKE studies. The red solid line in (**b**) is the fitting to the model described by Eq. (8).

On the other hand, and as observed in the hysteresis loops, the fitted data of *H*_*eff*_ shows a transition from positive values for *t*_*CoFeB*_ ≤ 4 Å, meaning that the multilayer films show perpendicular magnetization in the absence of external magnetic field, to negative ones (with in-plane magnetization) for *t*_*CoFeB*_ = 5 Å (See Fig. 4a). The sample with perpendicular magnetization and the larger positive *H*_*eff*_ values corresponds to the multilayer thin film with *t*_*CoFeB*_ = 3 Å. Also, we should note that the *H*_*eff*_ value for *t*_*CoFeB*_ = 1 Å is larger than for *t*_*CoFeB*_ ≥ 2 Å. This behavior, in combination with the fact that Co_20_Fe_60_B_20_, with a body-centered-cubic (bcc) crystalline structure, has a lattice parameter of 2.86 Å^79^, leads us to believe that both the 1 and 2 Å thick CoFeB layers are discontinuous thin films. Even, the continuity of the 3 Å thick CoFeB layers could be in question, which will be suggested below during the discussion related to the damping parameter. So for now, both thinner samples (*t*_*CoFeB*_ = 1 and 2 Å) were excluded in the subsequent analysis.

**Table 1:**
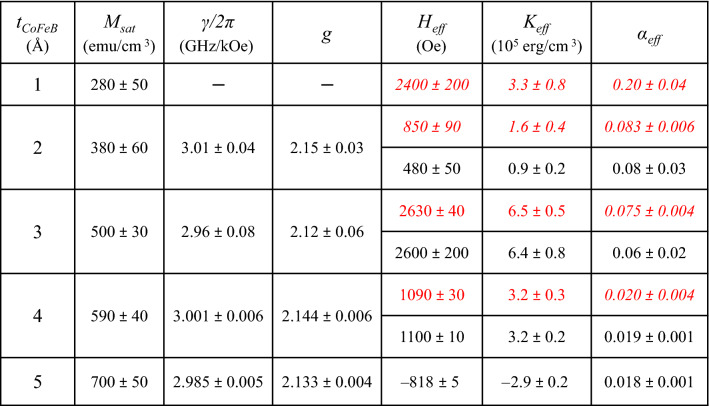
Summary of the results for [CoFeB/Pd]_N_ multilayer systems (*N* = *5* bilayers) as a function of the CoFeB thickness (*t*_*CoFeB*_): the saturation magnetization (*M*_*sat*_) extracted from VSM; the gyromagnetic ratio (*γ/2π*) and g-factor (*g*) determined from VNA-FMR fits using Eq. (4); Effective anisotropy field (*H*_*eff*_) determined from VNA-FMR (Eq. 4) and TR-MOKE (Eqs. 2, 3 and 5); Effective anisotropy energy (*K*_*eff*_) determined from Eq. (6); The effective damping (*α*_*eff*_) determined from VNA-FMR and TR-MOKE. Data extracted from the VNA-FMR measurements is shown in black. Data extracted from the *TR-MOKE* measurements is given in *italics* and *red*.

From the fitted data, the effective anisotropy energy (*K*_*eff*_) can be estimated according to (summarized in Table 1):

6 $$K_{eff} = \frac{1}{2}\left( {H_{eff} \times M_{sat} } \right)$$

Moreover, *K*_*eff*_ can be phenomenologically separated into a volume contribution *K*_*v*_ (erg/cm^3^) and a contribution from the interfaces *K*_*s*_ (erg/cm^2^), and it can be approximately described by the Néel model^80^:

7 $$K_{eff} = K_{v} + \left( {2K_{s} /t} \right)$$

where the origin of the factor of 2 in the last term is due to the presence of two identical interfaces per magnetic layer, and *t* is the magnetic layer thickness. Therefore, both *K*_*v*_ and *K*_*s*_ can be obtained by plotting (*t* × *K*_*eff*_) versus *t*. According to the Néel model^80^, (*t* × *K*_*eff*_) should show a linear dependence on *t,* where *K*_*v*_ is the linear dependence slope, and 2*K*_*s*_ corresponds to the intercept with the vertical axis. However, our data shows a deviation from the linear behavior at small CoFeB thicknesses (see Fig. 4b) as already observed in different systems like in Co/Au^81^ and Ni/Cu^82–84^ multilayer thin films. This deviation was explained using a phenomenological model that includes the shape anisotropy term, the bulk magnetocrystalline and magnetoelastic anisotropy energies, the surface magnetocrystalline and surface magnetoelastic anisotropy terms^83^ or the second-order of the magnetoelastic contribution^84^.

More recently, this non-linearity was also reported for NM/CoFeB/MgO thin films (where NM was Ta^69^ or Hf^23^) and it was fitted using the equation proposed by Gowtham et al*.*^69^:

8 $$K_{eff} \times t = K_{v}^{eff} \times t + 2 \times K_{s}^{eff} + \left( {K_{3} /t} \right)$$

where $K_{v}^{eff}$ is the effective volumetric contribution that should include the shape anisotropy term and both the bulk magnetocrystalline and bulk magnetoelastic anisotropy energy contributions; $K_{s}^{eff}$ is the effective surface term that includes the surface magnetocrystalline and magnetoelastic anisotropy terms as well as the second-order term of the bulk magnetoelastic contribution; and coefficient $K_{3}$ is related to the second-order term of the surface magnetoelastic contribution.

In agreement with Engel et al*.*^63^, we have estimated that (*t*_*CoFeB*_ x *K*_*eff_CoFeB*_) = (*t*_*CoFeB*+*Pd*_ x *K*_*eff*_) where *t*_*CoFeB*+*Pd*_ (*t*_*CoFeB*_) is the thickness of the CoFeB/Pd bilayer (CoFeB layer), *K*_*eff*_ is the measured effective anisotropy energy of the [CoFeB/Pd]_5_ system and *K*_*eff_CoFeB*_ is the effective anisotropy energy of a CoFeB thin film. Therefore, both *K*_*v_CoFeB*_ and *K*_*s_CoFeB*_ can be obtained by plotting (*t*_*CoFeB*_ x *K*_*eff_CoFeB*_) versus *t*_*CoFeB*_ (shown in Fig. 4b). Using Eq. (8) to fit the experimental data, we have estimated that $K_{v\_CoFeB} = \left( { - 16.2 \pm 0.3} \right) \times 10^{6} \;{\text{erg/cm}}^{3}$, $K_{s\_CoFeB} = \left( {0.53 \pm 0.01} \right) \;{\text{erg/cm}}^{2}$ and $K_{3} = \left( { - 1.48 \pm 0.05} \right) \times 10^{ - 8} \;{\text{erg/cm}}$. Our values are close to the data previously reported in the literature for Hf*/*CoFeB*/*MgO^23^ and Ta/CoFeB/MgO thin films^69^.

Assuming that the CoFeB layer is amorphous, its bulk magnetocrystalline anisotropy energy contribution should be null. Therefore, we suggest that the volumetric term could be formed by:

9 $$K_{v\_CoFeB} = K_{sh\_CoFeB} + K_{me\_CoFeB} = \left( { - 16.2 \pm 0.3} \right) \times 10^{6} erg/cm^{3}$$

where $K_{sh\_CoFeB} = - 2\pi M_{{eff_{CoFeB} }}^{2} = \left( { - 19 \pm 2} \right) \times 10^{6} \;{\text{erg/cm}}^{3}$ is the shape anisotropy term and $K_{me\_CoFeB} = \left( {3 \pm 1} \right) \times 10^{6} \;{\text{erg/cm}}^{3} \left( { = \left( {3 \pm 1} \right) \times 10^{5} \;{\text{J/m}}^{3} } \right)$ is the magnetoelastic anisotropy contribution.

As thin films are generally in a state of biaxial stress (*σ*_*x*_ = *σ*_*y*_ = *σ*_*in_plane*_ and *σ*_*z*_ = *σ*_*out_of_plane*_ = 0), the magnetoelastic anisotropy term can be defined by^18,85^:

10 $$K_{me\_CoFeB} = \frac{3}{2}\lambda_{s} \sigma_{in\_plane}$$

where *λ*_*s*_ and *σ*_*in_plane*_ are the saturation magnetostriction coefficient and the in-plane applied stresses, respectively. Moreover, *σ*_*in_plane*_ is related to the in-plane strain *ε*_*in_plane*_, via the Young's modulus (*E*) and the Poisson ratio (ν)^86^:

11 $$\sigma_{in\_plane} = \frac{{E\varepsilon_{in\_plane} }}{{\left( {1 - \nu } \right)}}$$

As the magnetoelastic anisotropy term favors that the magnetization lies perpendicular to the sample plane and the saturation magnetostriction coefficient of amorphous Fe_40_Co_40_B_20_ is positive *λ*_*s*_ = 20 × 10^–6^^87^, the magnetic layer should be under tensile stress with *σ*_*in_plane*_ ≈ (11 ± 3) × 10^10^ dyne/cm^2^ (≈ (11 ± 3) GPa). Using the Young's modulus *E* = 160 × 10^10^ dyne/cm^2^ (= 160 GPa) for CoFeB and the Poisson ratio *ν* = 1/3^88^, the analysis indicates a tensile in-plane strain of *ε*_*in_plane*_ ≈ (0.05 ± 0.01). This value is of the same order of magnitude than the strains reported by Gowtham et al*.*^69^ for Hf*/*Co_20_Fe_60_B_20_*/*MgO heterostructures with thicker CoFeB layers. The in-plane tensile strain in our ultrathin films should be related to the large lattice mismatch (≈36%)^18^ between Pd (3.88 Å)^89^ and CoFeB (2.86 Å for bcc)^79^ lattice parameters.

### Effective damping

We then studied the behaviour of the damping parameter (*α*) as a function of the CoFeB thin film thickness and it was extracted from the frequency linewidths (*Δf*_*FMR*_) in the VNA-FMR spectra. It was experimentally observed that the measured frequency linewidths (*Δf*_*FMR*_) are broadened by extrinsic contributions, which affect the calculations of the damping parameter^90^. Therefore, we used *Δf*_*FMR*_ to determine the apparent damping by^90^:

12 $$\alpha_{app} = \frac{{\Delta f_{FMR} }}{{\left( {\frac{\gamma }{2\pi }} \right)\left( {2H + H_{eff} } \right)}}$$

**Figure 5:**
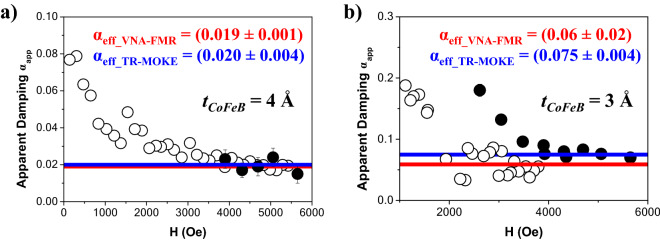
Apparent damping *α*_*app*_ as a function of the external applied magnetic field for CoFeB layer thickness of *t*_*CoFeB*_ = 4 (**a**) and 3 Å (**b**), extracted from VNA-FMR spectra (blavk open circle) and pump-probe measurements (black filled circle). The red (blue) solid lines are the average of *α*_*app*_ determined from the VNA-FMR spectra (pump-probe measurements) for large applied fields and correspond to the *α*_*eff*_.

Although *α*_*app*_ and the intrinsic damping may differ, we should note that *α*_*app*_ should give an upper limit for the intrinsic or Gilbert damping (*α*_*Gilbert*_). Open symbols in Fig. 5a and b show the typical curves of the apparent damping versus the external applied magnetic field for the multilayers with *t*_*CoFeB*_ = 4 and 3 Å, respectively. Although we cannot distinguish between the different extrinsic contributions to the linewidth, it is observed that *α*_*app*_ approaches a constant value for large applied fields. We have defined this value as the effective damping *α*_*eff*_ (see red lines in Figs. 5a and b).

Again, we should note that the signal-to-noise ratio is significantly low for those samples with the thinnest CoFeB thicknesses (*t*_*CoFeB*_ ≤ 3 Å), so optical studies were required to complete our analysis. From the pump-probe measurements, *α*_*app*_ can be determined using^73,91^:

13 $$\alpha_{app} = \frac{1}{{2\pi \times f_{FMR} \times \tau }} = \frac{1}{{\gamma \times \left( {H + H_{eff} } \right) \times \tau }}$$

where *f*_*FMR*_ is the resonance frequency and *τ* is the relaxation time. Both values were fitting parameters in Eq. (5) and they were used to analyze the precessional dynamics (as it was shown in Fig. 3b). Figure 5a and b show the apparent damping versus the external applied magnetic field for multilayers with *t*_*CoFeB*_ = 4 and 3 Å, respectively. Again *α*_*app*_ approaches a constant value for large applied fields and we assumed that this value is *α*_*eff*_ (see blue lines in Fig. 5a and b).

**Figure 6:**
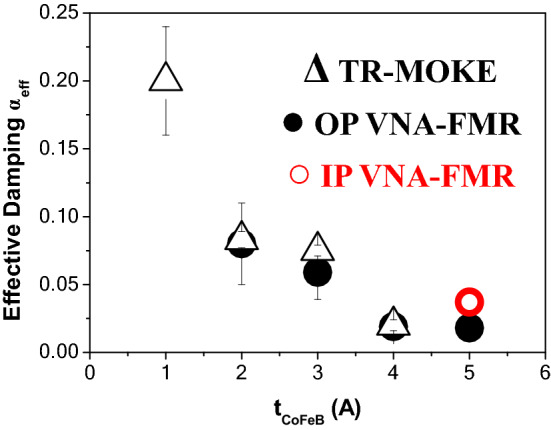
Evolution of the effective damping (*α*_*eff*_) versus *t*_*CoFeB*_ and determined from the TR-MOKE measurements (black open triangle), the perpendicular VNA-FMR (black filled circle) and the in-plane VNA-FMR (red open circle) measurements.

The evolution of the effective damping (*α*_*eff*_) vs. the CoFeB film thicknesses (*t*_*CoFeB*_) obtained by combining both types of measurements is summarized in Table 1 and shown in Fig. 6. It is important to note that both VNA-FMR and pump-probe have provided similar values. As one can see, samples may be separated into two groups: t_*CoFeB*_ ≤ 3 Å and t_*CoFeB*_ ≥ 4 Å. For the first group, the value of *α*_*eff*_ is large and decreases quickly with thickness increase. It reaches some kind of saturation at *t*_*CoFeB*_ = 4 Å and decreases only slightly (by 10%) for *t*_*CoFeB*_ = 5 Å. As Azzawi et al*.*^92^ reported in 2016, the non-continuity of thin films leads to the increase of the extrinsic contribution to the damping by adding an extra two-magnon scattering term. Therefore, we have suggested that the increase of *α*_*eff*_ for the thinnest CoFeB layers (t_*CoFeB*_ ≤ 3 Å) could be related to the abrupt rise of the two-magnon scattering contribution due to the non-continuity of the ultrathin layers.

To better understand the effective damping of the samples with nearly continuous CoFeB layers (*t*_*CoFeB*_ = 4 and 5 Å), we have tried to distinguish different contributions to the damping parameter that were briefly described in the introduction section. Remembering that eddy currents become important when the magnetic film thickness is comparable to or greater than the skin depth^90^, and the radiative damping is proportional to the magnetic layer thickness^57^, neither *α*_*eddy*_ nor *α*_*rad*_ should have significant contributions in our ultrathin films and they could be ignored. On the other hand, the literature suggests that the two-magnon scattering contribution (*α*_*TMS*_) is minimized in the perpendicular geometry, such as the one mainly used in this work^93^. In particular, Liu et al*.*^41^ studied the angular dependence of the two-magnon scattering contribution in a CoFeB thin film and confirmed that this effect is suppressed in the perpendicular configuration. They determined that the two-magnon contribution is significant when the sample magnetization angle, relative to the perpendicular direction or 0°, is pointed to an angle larger than 45°. Even *α*_*TMS*_ can achieve a value as large as the intrinsic damping in the in-plane configuration. Therefore, the *α*_*TMS*_ contribution could also be disregarded and the total measured damping should be mainly composed of the intrinsic term and the spin-pumping contribution:

14 $$\alpha_{eff} = \alpha_{Gilbert} + \alpha_{s - p}$$

The spin-pumping contribution *α*_*s-p*_ can be defined using the formula^94–96^:

15 $$\alpha_{s - p} = 2g\mu_{B} \frac{{g^{ \uparrow \downarrow } }}{{4\pi M_{eff\_CoFeB} }}\frac{1}{{t_{CoFeB} }}\left( {1 - e^{{ - 2t_{Pd} /\lambda_{Pd} }} } \right)$$

where *g* is the g-factor; µ_0_ = (9.27400915 × 10^–21^) erg/Oe is the Bohr magneton; *M*_*eff_CoFeB*_ = (1760 ± 80) emu/cm^3^ and *t*_*CoFeB*_ are the effective saturation magnetization moment and the thickness of the CoFeB layer, respectively; *g*^*↑↓*^ is the CoFeB/Pd interface spin mixing conductance; *t*_*Pd*_ = 0.5 nm is half the Pd layer thickness and *λ*_*Pd*_ = 9 nm^95^ is the spin diffusion length for Pd layer. The factor of 2 is related to the fact that the CoFeB layer is sandwiched by two Pd layers.

Considering the damping value for *t*_*CoFeB*_ = 4 Å (*α*_*eff*_ = (0.019 ± 0.001)) and assuming that *α*_*Gilbert*_ = 0.004^41,42,97^, we have estimated that *α*_*s-p*_ ≈ (0.015 ± 0.001). From Eq. (15), the CoFeB/Pd interface spin mixing conductance value should be *g*^*↑↓*^ ≈ (3 × 10^+15^) cm^−2^. Although this is a rough estimation, *g*^*↑↓*^ is of the same order of magnitude than the values already reported in the literature, particularly 2.21 × 10^+15^ cm^−2^ for a CoFeB/Pd interface^98^ or 0.722 × 10^+15^ cm^−2^ for a β-Ta/CoFeB interface^61^.

Finally, we estimated the two-magnon term (*α*_*TMS*_) for the sample with the most perfectly continuous CoFeB layers, namely the one with *t*_*CoFeB*_ = 5 Å. As it was mentioned above, *α*_*TMS*_ is negligible in the perpendicular geometry but can have an important contribution in the in-plane configuration. Therefore, for this sample we have compared the damping parameter, determined from in-plane VNA-FMR measurement (shown in Fig. 6) with the perpendicular one. While *α*_*eff*_^*OP*^ = (0.018 ± 0.001), the in-plane value is *α*_*eff*_^*IP*^ = (0.037 ± 0.008). Assuming that the difference between both values is due to the two-magnon contribution, we estimated that *α*_*TMS*_ = (0.019 ± 0.005), confirming the importance of *α*_*TMS*_ in the in-plane configuration and in agreement with literature^41^.

Summarizing, we have studied the magnetic behaviour of [CoFeB*/*Pd]_5_ multilayered thin films using two complementary techniques. The thicknesses of the CoFeB films reported in the literature have usually ranged between 0.5 nm to a few tens of nm. Here, we focused our attention in multilayers where *t*_*CoFeB*_ was varied between 1 and 5 Å. Our analysis determined an increase of the effective saturation magnetization from its bulk value to *M*_*eff_CoFeB*_ = (1760 ± 80) emu/cm^3^. PMA was observed in the as-cast samples for CoFeB layer thickness ≤ 4 Å. This behaviour was modeled by considering volumetric and surface anisotropy contributions. Then, we confirmed the presence of a strong surface anisotropy contribution as well as a magnetoelastic anisotropy term. This last term suggests that our ultrathin CoFeB films are under in-plane tensile strain. Finally, an effective damping parameter as low as *α*_*eff*_ ≈ (0.019 ± 0.001) was observed for the multilayer with 4 Å CoFeB thickness. We suggested that for *t*_*CoFeB*_ ≥ 4 Å the layer is continuous and the main contribution to effective damping is coming from spin-pumping when for lower thicknesses *α*_*eff*_ is dominated by two-magnon scattering.

## Methods

[CoFeB (*t*_*CoFeB*_)/Pd (10 Å)]_5_ multilayer thin films were sandwiched between a Ta(20 Å)/Pd(20 Å) seed bilayer and a 50 Å Pd capping layer. Samples were deposited at room temperature on Si(100) substrates using confocal dc magnetron sputtering with a base pressure below 2 × 10^−10^ Torr and an Ar working gas pressure of 5 mTorr (more details are given in Ref.^58^). The CoFeB layer thickness (*t*_*CoFeB*_) was varied between 1 and 5 Å and this alloy was sputtered from a Co_40_Fe_40_B_20_ target.

While room temperature magnetic hysteresis loops were measured in a vibrating sample magnetometer (VSM), the dynamical behaviour was studied by comparing TR-MOKE and VNA-FMR measurements. FMR measurements were carried out at room temperature using a coplanar waveguide (CPW) connected to a vector network analyzer. The samples were placed film down on the CPW, and the complex S_21_ parameter was measured as a function of the external magnetic field over a frequency range up to 20 GHz^99^. The external DC magnetic field (*H*) was applied along (*θ*_*H*_ = 90°) or perpendicular (*θ*_*H*_ = 0°) to the sample plane for in-plane and perpendicular measurements, respectively, and was always perpendicular to the ac field generated by the CPW. On the other hand, the dynamical response of the magneto-optical signals was obtained by an ultrafast pump-probe system based on a commercial Titanium:sapphire laser amplifier (Femtolasers Compact Pro CE-phase) delivering sub-30-fs laser pulses (approximately 40 nm bandwidth centered at 800 nm) with ~ 1 mJ of energy at a repetition rate of 1 kHz and with carrier-envelope phase (CEP) stabilization capability, seeded by a CEP-stabilised ultrafast laser oscillator (Femtolasers Rainbow). Time-resolved measurements were performed in polar MOKE configuration using 0.7% and 99.3% of the pulse energy for the probe and pump pulses, respectively. The spot size ratio between the pump and probe beams was adjusted to be 4:1 to assure that the probe spot hits a homogeneous pump illuminated area of the sample, and the pump fluence was fixed to 2 mJ/cm^2^. In order to increase the signal-to-noise ratio of the TR-MOKE signal, the external DC magnetic field was applied at an angle of *θ*_*H*_ = 78°^100^. All-optical measurements were performed under ambient conditions.
